# Protective Role of Fecal Microbiota Transplantation on Colitis and Colitis-Associated Colon Cancer in Mice Is Associated With Treg Cells

**DOI:** 10.3389/fmicb.2019.02498

**Published:** 2019-11-12

**Authors:** Zitao Wang, Wenjie Hua, Chen Li, Hao Chang, Ran Liu, Yangyue Ni, Hongzhi Sun, Yangyang Li, Xinyue Wang, Min Hou, Yu Liu, Zhipeng Xu, Minjun Ji

**Affiliations:** ^1^Department of Pathogen Biology, Nanjing Medical University, Nanjing, China; ^2^Department of Endocrinology, The Affiliated Sir Run Run Hospital of Nanjing Medical University, Nanjing, China; ^3^Jiangsu Province Key Laboratory of Modern Pathogen Biology, Nanjing Medical University, Nanjing, China

**Keywords:** colitis-associated cancer, fecal microbiota transplantation, regulatory T cells, gut microbiota, anti-inflammation

## Abstract

Colitis-associated cancer (CAC) is the most serious outcome of inflammatory bowel disease, which has an alteration of commensal intestinal microbiota. However, the role of intestinal microbiota on CAC progression is not well-understood. Fecal microbiota transplantation (FMT) was used for treating murine azoxymethane–dextran sodium sulfate (AOM-DSS) model of CAC. Composition of gut microbiota during FMT treatment was analyzed. RT-PCR and ELISA were used to detect the inflammatory factors, and immunofluorescence was applied to examine the phospho-nuclear factor (NF)-κB p65/p100 and Ki67-positive cells in the colons. In addition, flow cytometry was performed to analyze the immune cell after FMT treatment. Rehabilitation of the intestinal microbiota by FMT restored both the ratio and diversity of microbiota during CAC progression. Remarkably, a favorable morphometric outcome characterized by decreased tumor load and size was observed in CAC mice with FMT treatment. In addition, an anti-inflammatory function of FMT was demonstrated by decreasing pro-inflammatory factors but increasing anti-inflammatory factors through inhibiting canonical NF-κB activity and cellular proliferation in colons of CAC mice. The expression of CD4^+^CD25^+^Foxp3^+^ regulatory T cells (Tregs) was significantly increased after FMT treatment in CAC mice, but not T helper (Th)1/2/17 cells. Our study aids in the understanding of CAC pathogenesis and reveals a previously unrecognized role for FMT in the treatment of CAC through restoring the intestinal microbiota and inducing regulatory T cells.

## Introduction

Colorectal cancer (CRC) is one of the most common fatal malignancies globally (Weir et al., 2003). In China, the mortality rate attributable to CRC is estimated to reach 8.6 per 100,000 in 2020 (Zhu et al., 2017). The colitis-associated cancer (CAC), as one major subset of CRC, is closely related to chronic or dysregulated inflammation (Potack and Itzkowitz, 2008). It is urgent to clarify the mechanism underlying the development of CAC because about 20% of patients with chronic inflammation in the form of ulcerative colitis (UC) will develop CAC within 30 years from the onset, with at least half of the cases resulting in death (Yamamoto and Matsumoto, 2016).

Studies showed that inflammation is a critical cause for initiation and/or regulation of the progress of CAC (Kraus and Arber, 2009). Multiple cells that secrete proinflammatory cytokines or chemicals, that is, tumor necrosis factor (TNF)-α, interleukin (IL)-1, and IL-6, which can enhance cell proliferation and migration, thereby promoting tumor development (Grivennikov, 2013). However, IL-10 is a critical immunosuppressive cytokine that has been reported to link with inhibition of CAC (Mantovani et al., 2008; Uronis et al., 2009). The host immunity shapes a proper balance between pro- and anti-inflammatory response that is, respectively, mediated by T helper (Th) cells and T regulatory cells (Tregs). The excessive Th2/Th17 cells in intestinal mucosa are the main reasons for chronic inflammation and promote CAC progression (Amicarella et al., 2017). While Tregs, which are important in the limitation and regulation of immune responses, are believed to play an important role in maintaining gut homeostasis and reducing inflammatory responses (Salama et al., 2009; Erdman et al., 2010; van Herk and Te Velde, 2016).

A key player involved in the balance of pro- and anti-inflammatory processes is the gut microbiota (Gkouskou et al., 2014), which is popular in the intestinal tract and contains a myriad of bacterial strains that belong mainly to the phyla Firmicutes, Bacteroidetes, Proteobacteria, Actinobacteria, and Fusobacteria (Eckburg et al., 2005; Sender et al., 2016). Studies have also implicated that alteration in microbial composition and diversity is linked to CAC progression. The adherent/invasive *Escherichia coli* strains were highly abundant in the colonic mucosa of patients with colorectal carcinoma and adenoma (Cuevas-Ramos et al., 2010). *Fusobacteria* were enriched in colonic adenomas and in stool samples from patients with colorectal carcinomas (Kostic et al., 2013). Importantly, treatment with an antibiotic, which functions by depleting microbial ligands, worsened the severity of dextran sodium sulfate (DSS)-induced colitis in mice (Rakoff-Nahoum et al., 2004). Recently, an interesting study showed that the probiotics mixture of *Lactobacillus acidophilus, Lactobacillus rhamnosus*, and *Bifidobacterium bifidum* can reduce colitis in mice, which is accompanied by restoration of beta diversity but not alpha diversity of microbial species, suggesting that probiotics mixture could only partially change the component of the microbiota (Mendes et al., 2018). Therefore, alternative methods that can better restore the diversity of microbial species have emerged in the prevention of CAC.

Fecal microbiota transplantation (FMT) is one procedure that involves the complete restoration of the entire fecal microbiota instead of a single agent or combination of agents. FMT is being explored as a therapeutic strategy, aiming at the restoration of normal gut microbiota (Bakken et al., 2011). Recently, encouraging results have shown that using FMT is effective in the treatment of UC and Crohn's disease through altering dysregulated inflammation (Borody and Khoruts, 2011). However, the complex interaction between gut microbiota and antitumor immunity during CAC is not fully understood yet.

In this study, we demonstrated that FMT restored both the ratio and diversity of gut microbiota, which attenuated pro-inflammatory but promoted anti-inflammatory response through inducing Treg cells in CAC mice. Thus, we defined FMT as a potential novel therapeutic approach for CAC treatment.

## Results

### FMT Restored the Composition and Diversity of Gut Microbiota in the Colon

**Figure 1:**
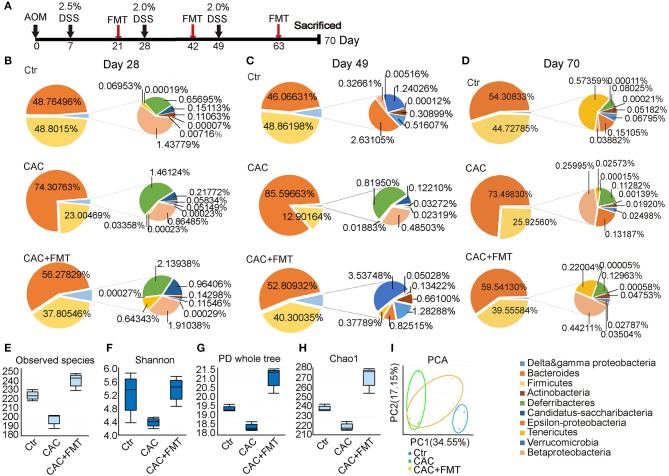
Changes of phyla at different time points and distribution in diversity. **(A)** Experimental protocol of azoxymethane–dextran sodium sulfate (AOM/DSS) model. Balb/c mice initially received a single intraperitoneal injection of AOM (7.5 mg/kg). One week after the AOM administration (set as Day 0). Mice in this model received 2.0% DSS in drinking water on day 7, 28, and 49 consecutive days. Stool samples were collected at the same day but before the DSS administration. FMT treatments are given on day 21, 42, and 63. Mice were then sacrificed on day 70 for further study. **(B**–**D)** Distribution of phyla at days 28, 49, and 70. Nine phyla (Actinobacteria, Bacteroidetes, Firmicutes, Beta-Proteobacteria, Delta and gamma-Proteobacteria, Epsilon-Proteobacteria, Deferribacteres Tenericutes, Verrucomicrobia) represented at least 99% of the reads. **(E**–**H)** Comparison of microbiota alpha-diversity on species richness **(E)**, Shannon Diversity Index **(F)**, Phylogenetic Diversity (PD) whole tree index **(G)**, and Chao1 Richness Index **(H)** in the fecal content of the cecum and colon from each group. **(I)** PCA test for the evaluation of β diversity. Mixed sample from each group, *n* = 6 per group.

To determine the impact of AOM/DSS protocol on mice's gut microbiota and whether FMT would interfere with its abundance and diversity, we analyzed the bacterial communities in fecal samples. Results showed that the abundance of phylum Firmicutes was obviously lower, but phylum Bacteroidetes was increased in CAC mice when compared to normal mice (Figures 1B,C). However, the abundance of the two major phyla was returned to normal level after FMT treatment, suggesting that the ratio of Firmicutes and Bacteroidetes was restored after FMT treatment and appeared in a donor-like manner (Figures 1B,D). In addition, the α-diversity of intestinal microbiota, as measured by the observed species, Shannon, PD Whole Tree index, and Chao1 index, was dramatically decreased in CAC mice; however, FMT treatment induced a statistically significant increase in the gut microbial composition (Figures 1E–H), indicating that the total number of the microbial species diversity was restored after FMT treatment. Furthermore, the fecal samples of CAC mice demonstrated a shift in clustering after FMT treatment, primarily along PC1, accounting for 34.55% of the intersample variation (Figure 1I), suggesting that the microbial community composition differs and was partially restored post-FMT treatment in CAC mice.

### FMT Suppresses CAC Tumorigenesis

**Figure 2:**
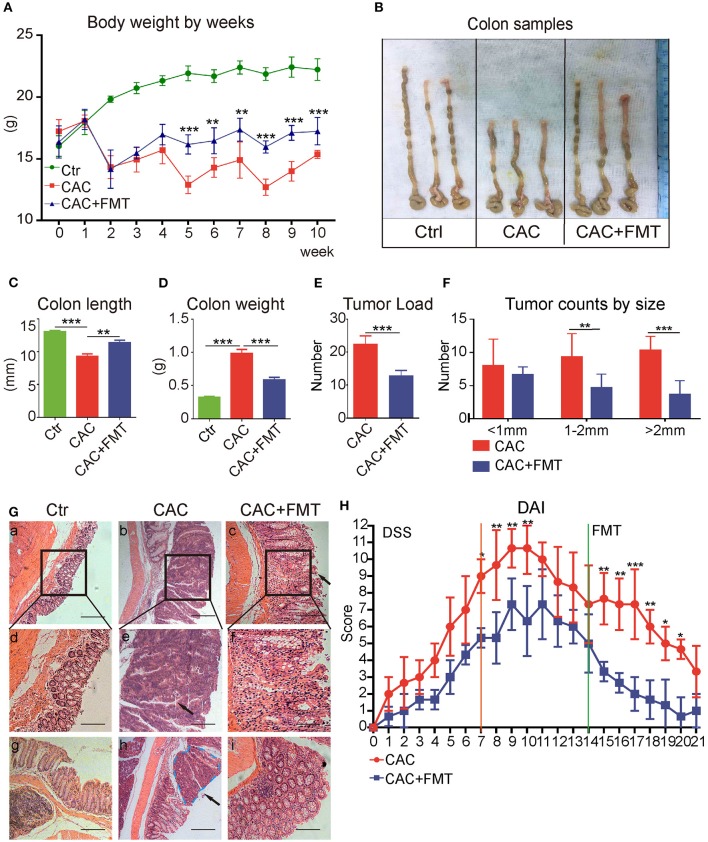
Effects of fecal microbiota transplantation (FMT) on azoxymethane–dextran sodium sulfate (AOM/DSS)-induced colitis and colon tumorigenesis in Balb/c mice. **(A)** FMT reduced DSS-induced body weight changes. The weights of mice in each group were examined. **(B)** Representative tumor and colon samples from each group. **(C–F)** The colon length changes **(C)**, colon weight **(D)**, tumor load **(E)**, and tumor size distribution **(F)** were investigated in each group. **(G)** The representative attached colon tissue (upper row), megascopic tumor/dysplasia area (mid row), and tumor/dysplasia boundary areas (lower row) from each group were shown by hematoxylin and eosin (H&E) staining. Bars in black represent size of 50 μm (20 μm for mid row). **(H)** FMT attenuated the experimental colitis, representative index was shown (second DSS cycle) expressed as Disease Activity Index (DAI), two-way ANOVA test. Data are expressed as the mean ± SD of six mice for each group, and the experiments were repeated three times with similar results. ***P < 0.001 [Student's *t*-test **(E)** or ANOVA/LSD **(A,C,D,F)**].

Thereafter, the role of gut microbiota in CAC mice was evaluated with FMT. As expected, we discovered that body weight loss in CAC mice was lessened after treatment with FMT (Figure 2A). In addition, the colon length, which is referred to as a sign of colon inflammation and denoted the progression and severity of damage of the colon (Neufert et al., 2007), was significantly decreased in CAC mice when compared with normal mice. However, our data consistently manifested a significant increase in intestinal length of FMT-treated mice in comparison with CAC mice (Figures 2B,C). We also found that FMT treatment resulted in a significant reduction in overall colon weight and tumor counts (Figures 2D,E). Notably, although there were no significant differences in the number of small polyps (<1 mm), a statistically significant decrease was observed in the number of middle (1–2 mm) or large polyps (>2 mm) in CAC mice under FMT treatment (Figure 2F). Furthermore, the histological study of the colon in the CAC mice revealed extensive chronic inflammation, with numerous and large tumors constituted by atypical epithelial cells with dysplastic nucleus. In contrast, less inflammation, mild dysplasia, and relative normal structure of colon gland in FMT-treated mice were observed (Figure 2G). Disease Activity Index (DAI) scores also suggested a decreased disease symptom and alleviated inflammatory responses after FMT treatment (Figure 2H). These results suggest that FMT protects against intestinal carcinogenesis induced by AOM/DSS.

### FMT Regulated Colon Inflammation Associated With Progression of CAC

**Figure 3:**
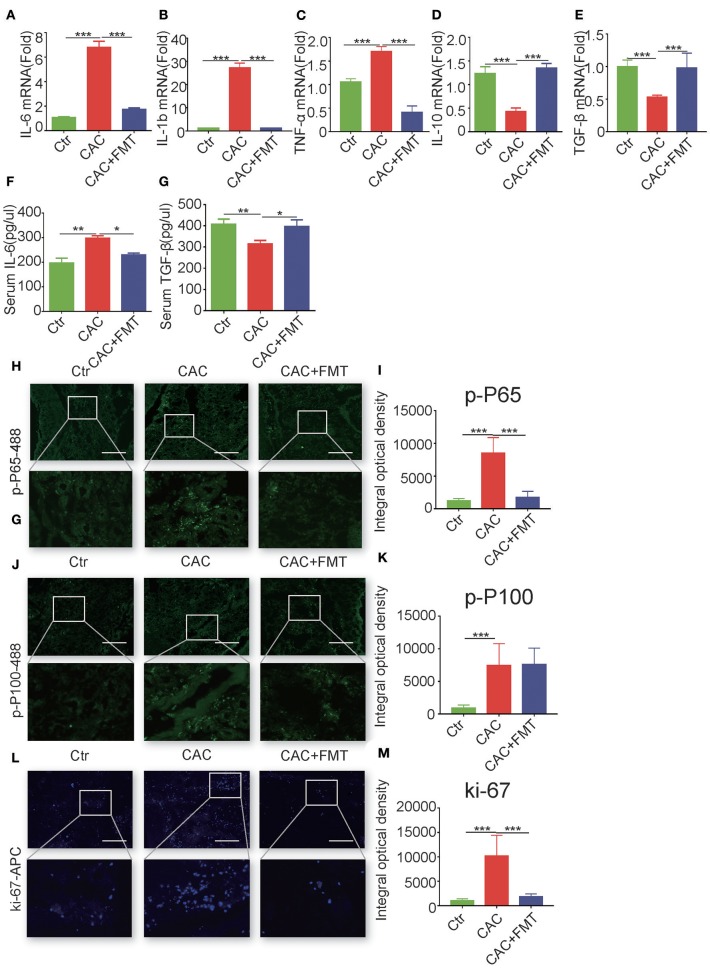
Fecal microbiota transplantation (FMT) downregulates pro-inflammatory cytokines and promote anti-inflammatory responses and inhibits nuclear factor (NF)-κB activity and cellular proliferation. **(A–E)** Real-time RT-PCR analysis of interleukin (IL)-6 **(A)**, IL-1β **(B)**, tumor necrosis factor (TNF)-α **(C)**, IL-10 **(D)**, transforming growth factor (TGF)-β1 **(E)** in colon tumor tissue of each group; results are presented using the 2—ΔΔCt method (ANOVA/LSD). **(F,G)** Concentration of serum cytokines TGF-β and IL-6 analyzed by ELISA at day 70 after being injected with azoxymethane (AOM) (ANOVA/LSD). Immunofluorescence of Pho-p65 **(H)**, pho-p100 **(J)**, or Ki67 **(L)** in the colon tissue from CAC mice treated with or without FMT. Original magnification, ×200. Bars in white represent size of 50 μm (20 μm for mid row). The mean optical density of Pho-p65 **(I)**, Pho-p100 **(K)**, or Ki67 **(M)** positive cells from 10 random fields in each mouse was digitized and analyzed on Image-Pro Plus software. Data are expressed as the mean ± SD of six mice for each group, and the experiments were repeated three times with similar results. *P < 0.05, **P < 0.01, ***P < 0.001.

We next evaluated whether the antitumor activity of FMT was associated with its anti-inflammatory properties. As shown in Figure 3, the mRNA levels of pro-inflammatory IL-1β, IL-6, and TNF-α were markedly elevated in the colon tissues of AOM/DSS-induced mice, relative to that in the control group (Figures 3A–C). However, the levels of IL-1β, IL-6, and TNF-α in the colon tissues were significantly inhibited by FMT treatment (Figures 3A–C). In addition, dramatically increased mRNA levels of anti-inflammatory cytokines IL-10 and transforming growth factor (TGF)-β were observed in colonic tissues of FMT-treated CAC mice (Figures 3D,E). Consistent with this, the serum level of IL-6 was markedly enhanced, but TGF-β expression was decreased in AOM/DSS-induced mice; however, treatment with FMT regulated the balance of IL-6 and TGF-β levels (Figures 3F,G). Altogether, these results indicate that FMT induces anti-inflammatory response and reduces pro-inflammatory response in AOM/DSS-induced CAC mice.

### FMT Inhibited Canonical NF-κB Activity and Cellular Proliferation in Colons From CAC Mice

Given that NF-κB activation, which is composed of canonical pathway and non-canonical pathway, plays a critical role in the regulation of colon inflammatory cytokines (Sun, 2012), we next investigated the expression of canonical phospho-NF-κB p65 (Ser536) and non-canonical phospho-NF-κB p100 (Ser866/870) in the colon tissues of CAC mice with or without FMT treatment. Results showed that phospho-p65-positive infiltrating immune cells were markedly elevated in colons of AOM/DSS-induced CAC mice but were substantially reduced by FMT treatment (Figures 3H,I). Although the expression of phospho-p100 was increased in colons of CAC mice, no effect of FMT on non-canonical NF-κB signaling (p100 phosphorylation) was found (Figures 3J,K).

Considering the centrality of NF-κB signaling in promoting cellular proliferation and survival (Karin and Greten, 2005), we evaluated the distribution of Ki67-positive cells in actively inflamed and neoplastic regions of colons isolated from CAC mice with or without FMT treatment. Results showed that the colonic mucosa from CAC mice increased proliferation when compared with normal mice; however, FMT inhibited the numbers of Ki67-positive cells (Figures 3L,M). Taken together, these data suggest that the weakened inflammatory and proliferative state observed in the context of the FMT-treated CAC mouse colon may contribute to the protective roles against intestinal carcinogenesis induced by AOM/DSS.

### FMT Treatment Enhances CD4^+^T Cell Response in Colon Lamina Propria Lymphocyte (LPL)

**Figure 4:**
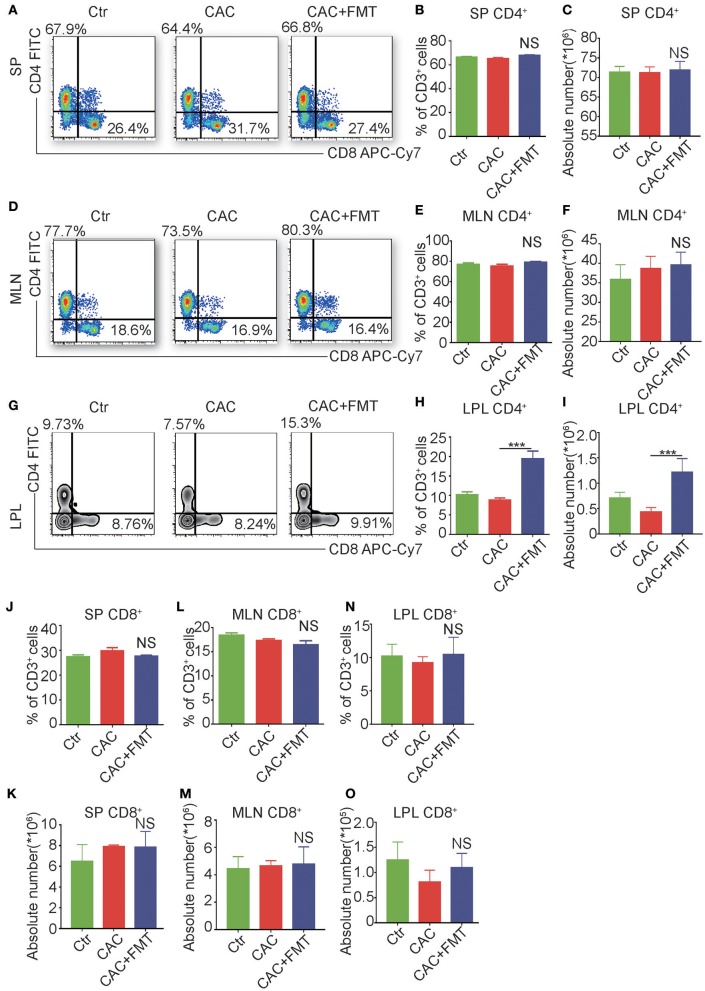
Fecal microbiota transplantation (FMT) treatment promotes CD4^+^ T cells but not CD8^+^ T cells in lamina propia lymphocytes (LPLs). **(A–O)** Single-cell suspensions of mouse LPL, mesenteric lymph nodes (MLN), and spleens from each group were prepared. Cells were stained with CD3-Percp-Cy5.5, CD4-FITC, CD8-APC-Cy7 for FACS analysis of CD3^+^CD4^+^ T cells (Th cells) or CD3^+^CD8^+^ T cells (Tc cells). Representative FACS gates from spleen **(A)**, MLN **(D)**, and LPL **(G)** are presented. The proportion and absolute numbers of Th cells **(B,C,E,F,H,I)** or Tc cells **(J–O)** in CD3^+^ T cells from splenocytes **(B,C,J,K)**, MLN **(E,F,L,M)**, and LPL **(H,I,N,O)** from each group were analyzed by FACS. Data are expressed as the mean ± SD of six mice for each group from one representative experiment. All experiments were repeated three times with similar results. ***P < 0.001 (ANOVA/LSD).

It is reported that adaptive immune cell is highly involved in the regulation of the inflammation and is a major contributor of inflammatory cytokines (Antoniou et al., 2015). We further examined the adaptive immune cells in each group. As shown in Figure 4, although there was no significant difference of CD3^+^CD4^+^ T cells in spleen, mesenteric lymph nodes (MLN), and FMT-treated CAC mice (Figures 4A–F), dramatic increases both in the percentage and absolute number of CD3^+^CD4^+^ T cells in LP were observed in CAC mice after receiving FMT treatment (Figures 4G–I). In addition, the expression of CD3^+^CD8^+^ T cells in the spleen, MLN, and LP revealed little change but not statistically significant after FMT treatment (Figures 4J–O). These data corroborate that FMT treatment attenuates intestinal carcinogenesis, and this may be associated with the increased CD4^+^ T cell response in intestinal LP of CAC mice.

### FMT Triggers the Accumulation of CD4^+^CD25^+^Foxp3^+^ Treg Cells in CAC Mice

**Figure 5:**
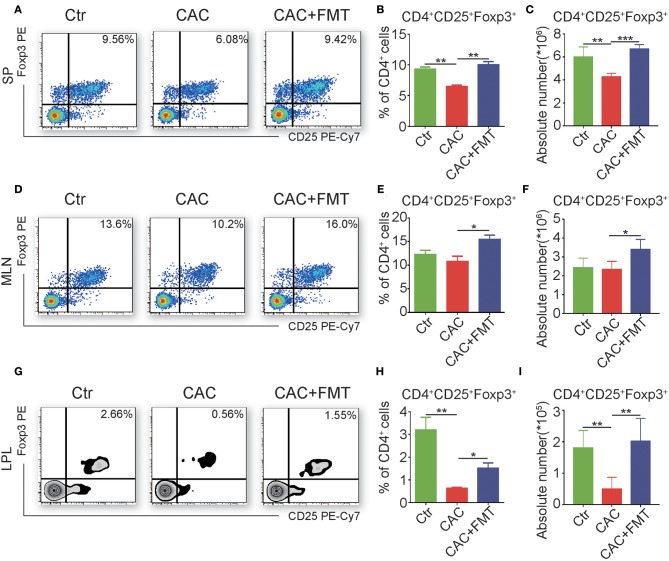
Fecal microbiota transplantation (FMT) promotes regulatory T (Treg) cell responses. **(A–I)** Single-cell suspensions of mouse lamina propria lymphocyte (LPL), mesenteric lymph node (MLN), and spleens from each group were prepared. Cells were stained with CD4-FITC, CD25-PE-Cy7, and then intracellularly stained with phycoerythrin (PE)-conjugated antibodies against Foxp3 for fluorescence-activated cell sorting (FACS) analysis of Treg cells. Representative FACS gates from spleen **(A–C)**, MLN **(D–F)**, and LPL **(G–I)** are presented. The proportion **(B,C,H)** and absolute numbers **(C,F,I)** of Treg cells in CD4^+^ T cells from splenocytes **(A–C)**, MLN **(D–F)**, and LPL **(G–I)** from each group were analyzed by FACS. Data are expressed as the mean ± SD of six mice for each group from one representative experiment. All experiments were repeated three times with similar results. *P < 0.05, **P < 0.01, ***P < 0.001 (ANOVA/LSD).

To further elucidate the cellular mechanism underlying the antitumor effect of FMT, we investigated the CD4^+^ T cell subsets to understand whether FMT treatment affects CD4^+^ T cell differentiation. Results showed that slight but not significantly changed percentages and absolute cell numbers of Th1, Th2, and Th17 cells were observed in the FMT-treated CAC mice when compared to untreated CAC mice (Figures S1–S4). However, Treg cells were significantly upregulated in spleen, MLN, and LPL of CAC mice after FMT treatment. These phenomena suggest that FMT may induce the accumulation of Treg cells, which could alleviate inflammatory responses in CAC mice (Figure 5). To be noticed, an increased percentage of ST2^+^ Treg cells was found after FMT treatment in LPL. In addition, the expression of CD25, which is one marker of antigen-specific Tregs (Chen et al., 2003), in Treg cells was decreased in the CAC group while recovered by FMT treatment (Figure S5). This suggests that the role of ST2^+^ Treg cells in the protection of CAC may be crucial. In addition, the expression of Tc1 cells, which were critical for cytotoxic effect against tumor cells (Kemp and Ronchese, 2001), showed only slight changes after FMT treatment in CAC mice (Figure S6).

### Fecal Microbiota or Butyrate Induced CD4^+^CD25^+^Foxp3^+^ Treg Cells *in vitro*

**Figure 6:**
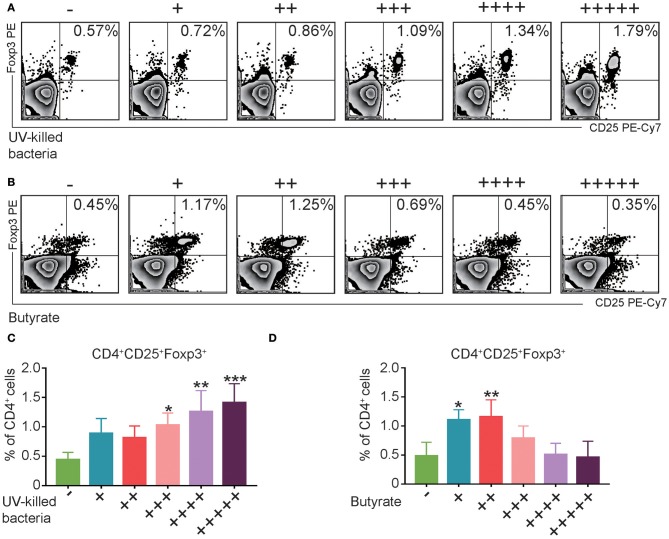
UV-Killed bacteria and butyrate induce regulatory T (Treg) cells *in vitro*. **(A,C)** Splenocytes from control Balb/c mice were purified, and then co-cultured with UV-killed bacteria obtained from mice's feces for 12 h, and then the percentages of Treg cells were analyzed by FACS. **(B,D)** Splenocytes from control Balb/c mice were purified and then co-cultured with butyrate in different concentration (+31.25, ++62.5, + + +125, ++ + +250, and + + + + +500 μM) for 12 h, and then the percentages of Treg cells were analyzed by FACS. Data are representative of three independent experiments with similar results. *P < 0.05, **P < 0.01, ***P < 0.001 (ANOVA/LSD).

Consistent with the *in vivo* data, *in vitro* treatment of splenocytes from normal mice with UV-killed fecal microbiota resulted in a dose-dependent increase in Treg cells (Figures 6A,C). However, butyrate, which is the major metabolite of phylum Firmicutes (Louis and Flint, 2009), induced the expression of Treg cells only at the low levels (Figures 6B,D). Altogether, these data suggest that fecal microbiota and the representative product butyrate may contribute to the induction of Treg cells in FMT-treated mice.

## Discussion

Although it has been well-accepted that the role of modifying gut microbiota in treating inflammatory bowel diseases (IBD) is crucial, it remains largely unclear whether FMT could associate with gut carcinogenesis (Zitvogel et al., 2017). The present study demonstrated that FMT attenuated the CAC progression by modulating the interaction between microbiome and host immune, thus favoring Treg cells and reducing gut inflammation.

CAC is one of the most common types of cancer that causes massive mortality (Brenner et al., 2014). The mechanism for tumor initiation and promotion is complex. It is generally recognized that inflammation plays a critical role in the development of CAC (Grivennikov, 2013). For IBD patients who have greater severity and extensive colitis, the risk to develop CAC is pronounced (Itzkowitz and Harpaz, 2004). The tumors tend to grow at the sites of chronic inflammation with overexpression of pro-inflammatory cytokines IL-6, COX-2, and TNF-α in human CAC. Non-steroidal anti-inflammatory drugs (NSAIDs) have long been applied in treating CAC to reduce mortality (Kraus and Arber, 2009). The upregulation of these cytokines is closely associated with gastrointestinal tumor formation (Li et al., 2012). Popivanova et al. have recently shown that blockade of TNF-α reduces the formation of colorectal tumors in mice lacking the TNF receptor. These findings suggest that blocking of TNF signaling can reverse tumorigenesis even when CRC is already present (Popivanova et al., 2008). Another crucial cytokine involved is IL-6. DSS-induced mucosal inflammation or colitis was markedly increased in both IL-6-deficient mice and in mice lacking STAT3 in intestinal epithelial cells (Kraus and Arber, 2009). Altogether, these studies suggest that effective interventions for inflammation might be a benefit for CAC treatment. Previously, researchers found that the interplay between host immune factors and the intestinal microbiota accounts for the abnormal inflammatory response observed in IBD patients (Sands, 2007). In this study, we discovered an altered gut microbiome composition in CAC models characterized by unbalanced Firmicutes and Bacteroides. Meanwhile, the overall diversity of microbiome was also decreased. This is consistent with the report that the inflammation and cancer development modify the microbiome in multiple content (Yamamoto and Matsumoto, 2016).

To modify gut microbiota may have a critical role in the treatment of CAC. Previous studies had introduced single or several bacteria complex strains to treat CAC (Lenoir et al., 2016; Mendes et al., 2018). But these approaches showed less effectiveness in rescuing the diversity of microbiota that proved to be crucial to gut homeostasis (Sanchez et al., 2017). Therefore, we used FMT, which is among the novel clinical interventions that target gut microbiome, to treat CAC. As reported, FMT took advantage of a well-balanced ecosystem being transferred from donors to another, thus increasing the possibility of a long-term reset of the microbiome (Zitvogel et al., 2017). In our study, we found a restored shift in the ratio of Bacteroidetes to Firmicutes species after FMT treatment. It also showed superiority in restoring gut microbiota diversity. Indeed, the number of species observed in FMT-treated CAC mice is even higher than that of the control group. This could partly be due to the administration of AOM/DSS, which had changed the composition of the fecal microbiota with newly developed species or columns. Even the general diversity of microbiota was decreased in the CAC group, the composition can be largely different from that of the control group. Thus, by the treatment with FMT, the overall composition can be an interaction of “good and evil” and revealed in a manner of increased numbers of species even compared to control by the time we examined the fecal sample. Previous research also report a healthier and longer life in humans with a more diverse gut microbiota (Valdes et al., 2018), suggesting that an increase in diversity may be a favorable outcome. As expected, our data also found that FMT administration modulates gut inflammation and results in a significant reduction in overall polyp number and size. To the best of our knowledge, no other study has evaluated the impact of the association of FMT on the richness, diversity, and abundance of the colon microbiota in CAC.

Several studies have highlighted the effect of the IL-6/Janus Kinase/Signal transducers and activators of transcription (JAK/STAT) signaling pathway on cancer initiation and progression (Hodge et al., 2005). Our results discovered an increase of these pro-inflammatory cytokines in CAC mice but were decreased after FMT treatment. Moreover, FMT-treated mice promoted the production of anti-inflammatory IL-10 and TGF-β in CAC mice. However, we were not able to detect the sources of the inflammatory cytokines and the cellular mechanism in the gut because of the limited number of immune cells that could be collected from colonic LPL of each mouse. As NF-κB signaling activation plays a pivotal role in colitis-associated CRC development (Sun, 2012), we detected its activation and found that FMT treatment reduced the colonic expression of phospho-p65, which is the key factor of canonical NF-κB pathway regulating colon inflammations (Dijkstra et al., 2002), but did not influence phospho-p100 in AOM/DSS-induced CAC mice, suggesting that the whole gut microbiome mainly activated the canonical NF-κB pathway in the colon. The critical roles in promoting cellular proliferation and survival of NF-κB activation had been reported (Karin and Greten, 2005). Araki et al. (2010) found that the proliferation of colonic epithelium was decreased in AOM/DSS-induced CAC mice, and our data showed that FMT inhibited the proliferation, as determined by Ki-67 staining, in the colons from AOM/DSS-induced CAC mice, indicating the attenuated proliferative state in colons of FMT-treated CAC mice involved in the protective roles against intestinal carcinogenesis.

Adaptive immune cells are major contributors and regulators of pro- and anti-inflammatory responses in CAC (Antoniou et al., 2015). Studies showed that high infiltrates of Tregs have been associated with a positive outcome of patients in a minority of cancers including bladder, colorectal, and esophageal (Mantovani et al., 2008; Kraus and Arber, 2009). Although the exact function of Treg cells in these tumor remains unknown, the inflammation-suppressive effect of Treg cells by producing IL-10 is widely believed to reduce the risk of CAC (van Herk and Te Velde, 2016). To provide a better understanding of gut immune response in CAC, we had detected the level of Th1/Th17 and CD8 T cells in the MLN, the major gut-associated lymphoid tissue serves as a firewall in the central gut immune system (Macpherson and Smith, 2006). Importantly, previous study had confirmed that DSS-induced colitis is closely correlated with the functional status of the MLN (Spahn et al., 2002). In our results, we detected the changes in the mentioned T cell levels in MLN and found that there were no significant changes in CAC mice after FMT treatment. So, we focused our attention on the more pronounced Treg cells. In our study, an upregulation of Treg cells was found in CAC mice after FMT treatment, suggesting that the weakened tumor progression during FMT treatment was mainly dependent on the induction of Treg cells in CAC mice. The protective role of ST2/IL-33 in the setting of IBD has well-been recognized (Griesenauer and Paczesny, 2017). Previous research has shown that Tregs in colon preferentially express ST2 that promotes Treg accumulation and their protective function against inflammation through ST2/IL-33 (Schiering et al., 2014). In our findings, the cell percentage of ST2^+^ Treg cells was decreased in CAC. However, following the FMT treatment, we discovered increased ST2^+^ Treg in LPL and also spleen, which suggests that the protective role of Treg cells could possibly be related to ST2/IL-33 signaling.

Interestingly, the inducement of colon Treg cells is closely related to the gut microbiome. Reports showed that Treg cells can be upregulated by certain bacterial strains and metabolic substances from phylum Firmicutes (Nagano et al., 2012; Nicholson et al., 2012). In addition, butyrate is believed to have a potential protective role in colorectal colitis or CAC since it is capable of inducing Treg cells and anti-inflammatory response in a Ffar2 (GPR43)-dependent manner (Zhang et al., 2016). The treatment of butyrate can increase Helio^+^ Treg numbers in GF mice, specifically Foxp3^+^ IL-10-producing Tregs (Grivennikov, 2013). Treatment of PSA as a substitute to live microbiota could induce Treg cells that include higher basal levels of GITR, IL-10, ICOS, CTLA-4, TGF-β_2_, and Ebi3 (Round and Mazmanian, 2010). Our study showed that butyrate seemed to have less effect on induction of Treg cells when compared with entire flora *in vitro*, which suggested that the anti-inflammatory effect was mediated mainly through the complex microbiome.

There remain some limitations to mention in this work. Our result was achieved through murine models and has certain differences compared to FMT in human. Clinically, FMT was performed under colonoscopy or through nasal tubes to reach specific regions of diseases, which maximized the clinical effectiveness. While we mimic this procedure through oral gavage, although it has advantages of quantity control and simple procedure, it does leave the microbial fluid in the acid gastric environment, which could influence the outcome of FMT. Although the general diversity was recovered, the microbial load in the colon remained far less. Also, due to the limitation of current testing methods, we were not able to continually monitor the dynamic and interactive changes of gut microbiota, which indeed require further researches.

In summary, this study demonstrates the reinforcement of natural defenses and protection against gastrointestinal disorders during CAC development. Moreover, we provide evidence to show that FMT could enhance anticancer immunity through inducing Treg cells in the microenvironment of tumors (Figure S7). Our data suggest a promising therapeutic strategy for FMT in the treatment of intestinal cancer, although the safety and ethical concern behind FMT treatment still needs further researches (Borody and Khoruts, 2011).

## Materials and Methods

### Mice

Six-week-old female Balb/c mice were obtained from Animal Core Facility of Nanjing Medical University and bred under SPF conditions. Mice were weighed and distributed equally into three groups (*n* = 6) labeled with Ctr, CAC, and CAC + FMT. All experiments were performed in line with the Regulations for the Administration of Affairs Concerning Experimental Animals (1988.11.1). Animal procedures were approved by the Institutional Animal Care and Use Committee (IACUC) of Nanjing Medical University for the use of laboratory animals.

### CAC Mice Model Establishment

CAC was induced using a well-established protocol as described (Neufert et al., 2007). Briefly, AOM (Sigma, cat. no. A2853) solution (10 mg AOM per 1 kg mice) was injected intraperitoneally into mice at days 1 and 3 circles of 2.5% DSS (MP Biomedicals) in drinking water (7 days DSS and 14 days water) to induce a long-lasting chronic DSS colitis in these mice. Control group was injected with the same volume of sterile isotonic saline and fed with normal drinking water. All groups consumed equal amounts of total DSS or drinking water (data not shown). Mice were sacrificed at day 70 for analysis. The 10-week-protocol used to induce CAC is presented in Figure 1A. The black arrows indicate the dates when DSS was administered, and red arrows indicate the dates when FMT treatments were performed.

### Fecal Microbiota Transplantation

Fecal microbiota transplantation was conducted as previously described with a few modifications (Ekmekciu et al., 2017). Briefly, fresh feces were collected from 10 age- and gender-matched mice and homogenized in 10 ml of sterile PBS and centrifuged for 30 s at 3,000 rpm, 4°C, to pellet the particulate matter. OD value of the supernatant slurry was checked to calculate the concentration of total bacteria (OD = 0.5 represents 10^8^ cells). For each mouse, 1 × 10^8^ bacterial cells (sum of total bacterial population within 0.2 g fecal contents) were centrifuged for 5 min at 12,000 rpm, 4°C. The bacterial pellets were resuspended in 0.2 ml PBS and gavaged into each mouse at days 14, 35, and 56. Mice from group Ctr and CAC were gavaged with 0.2 ml of PBS.

### Quantitative Analysis of Bacterial Communities

Mice from different groups were separated for feces collection. The total genomic DNA from each fecal sample (200 mg) was extracted using a TIAamp DNA stool mini-kit according to the manufacturer's instructions, resuspended in 40 ml of TE buffer (pH 8.0) and quantified with an Eppendorf Nanodrop. These samples were analyzed with the qPCR assay (primers used were listed in Table S1), and the Ct values were used to calculate the proportion of bacterial taxa in the feces as described previously (Yang et al., 2015).

### Deep Sequencing of 16S rRNA Genes

Before extraction, fecal samples collected from each group at each time point were mixed and homogenized. Fecal samples were mixed with 1 ml of extraction buffer [50 mM Tris (pH 7.4), 100 mM EDTA (pH 8.0), 400 mM NaCl, and 0.5% SDS containing 20 μl proteinase K (20 mg/ml)]. Total genomic DNA from fecal samples (200 mg) was extracted using the TIAamp DNA stool mini-kit according to the manufacturer's instructions. The forward primer (5′-ACTCCTACGGGAGGCAGCAG-3′) and the reverse primer (5′-GGACTACHVGGGTWTCTAAT-3′) containing the A and B sequencing adaptors (454 Life Sciences) were used to amplify a region covering the V3–V4 region of the 16S rRNA gene. PCRs were carried out in triplicate using 25-μl reaction mixtures with 5 μM concentrations of each primer, 30 ng of template DNA, 5 μl of the PCR buffer, and 12.5 μl of 2× Taq Plus Master Mix. The amplification program consisted of an initial denaturation step at 94°C for 5 min, followed by 28 cycles of 94°C for 30 s (denaturation), 55°C for 30 s (annealing), 72°C for 60 s (extension), and then a final extension of 72°C for 7 min. Replicated PCR products of the same sample were mixed and purified with a DNA gel extraction kit. Three PCR products per sample were pooled to mitigate reaction-level PCR biases. The PCR products were purified using a QIAquick Gel Extraction Kit (QIAGEN, Germany), quantified using Real Time PCR, and sequenced at Allwegene Company, Beijing.

### Sequence Processing and Bioinformatics Analysis

Deep sequencing was performed on Miseq platform at Allwegene Company (Beijing). After the run, Illumina Analysis Pipeline Version 2.6 was used to perform image analysis, basic calls, and error estimation. After raw data were screened first, sequences were removed if they accorded with one of the following characters: shorter than 200 bp, containing ambiguous bases, having a low-quality score (≤20), or incomplete match to primer sequences or bar code tags. The sample-specific bar code sequences were applied to separate qualified reads, which were subsequently trimmed with Illumina Analysis Pipeline Version 2.6. Then the data set was analyzed via QIIME. (The data set is available at: https://dataview.ncbi.nlm.nih.gov/?search=SUB6299086). To generate rarefaction curves and to calculate the richness and diversity index, the sequences were clustered into operational taxonomic units (OTUs) with a 97% similarity (Cole et al., 2009). All sequences into different taxonomic groups were classified by the Ribosomal Database Project (RDP) Classifier tool (Wang et al., 2008). Using the OTU table to normalize the smallest sample size, α- and β-diversity analyses were performed *via* the R package Phyloseq v.1.19.1 and vegan 2.4.2 packets (McMurdie and Holmes, 2014). Kruskal-Wallis test was used to identify differential-abundant taxa between groups (*P* < 0.05). The bar plot shows the taxa with relative abundance >1%. To examine the similarity between different samples, PCA were applied via R on the basis of OTU information from each sample.

### Symptom Severity

Symptom score was evaluated as previously described with some modifications (Ghia et al., 2006). Briefly, animal health was monitored by adding the scores of weights loss (WL) (>5% WL = 0; 6–10% WL = 1; 11–15% WL = 2), fecal consistency (pellet = 0; pasty = 2; liquid = 4), and blood in stools (assessed with negative = 0, positive = 2, and gross bleeding = 4). DAI was scored during each DSS treatment cycle.

### Histology

Paraffin tissue block sections of the distal colons were stained with H&E, subsequently evaluated blindly by a pathologist (I.C.) and characterized according to the severity of tumor dysplasia when a tumor was present in the section: low-grade dysplasia (LGD) or high-grade dysplasia (HGD).

### Real-Time PCR

RNA was derived with an RNeasy Mini Kit (cat: 74104; Qiagen, Hilden, Germany) and measured using a BioPhotometer (Eppendorf, Hamburg, Germany). cDNA was synthesized from 2 μg of RNA with a RevertAid First strand cDNA Synthesis Kit (cat: K1621; Fermantas Life Sciences, St. Leon-Rot, Baden-Württemberg, Germany). Power SYBR Green PCR Master Mix (cat: 4309155; Applied Biosystems, Foster City, CA) was used to carry out real-time PCR via the 7300HT Fast Real-Time System (Applied Biosystems) according to the protocol. The sequences of gene-specific primers were published previously (Table S1). SDS software (Applied Biosystems) was used to analyze data. Results were normalized to the expression of GAPDH.

The cycling parameters contained three stages: stage one, at 50°C for 2 min; stage two, at 95°C for 10 min; stage three, 40 cycles of 95°C for 15 s and 60°C for 1 min, the melting curve analysis process included. Expressions of mRNA were calculated using a comparative method (2^−ΔΔCt^).

### ELISA and Serum Analysis

Levels of IL-6 and TGF-β in serum were quantified with an ELISA kit (Dakewe Biotech Co., Ltd.) according to the instructions provided by the manufacturer.

### Isolation of Splenocytes, MLN, and LPL

Briefly, spleens and MLN were minced for preparation of splenocytes and lymphocytes. Splenocytes were then rinsed with an erythrocyte lysis buffer, meshed through a 100-mm cell strainer, and washed with PBS containing 2 mmol/L EDTA and 2% fetal calf serum (FCS). MLN cells were meshed through a 100-mm cell strainer and washed with PBS containing 2 mmol/L EDTA and 2% FCS. LPL were isolated as described previously (Neufert et al., 2007). Briefly, colons were washed with PBS to remove feces, cut open longitudinally, and cut into 1-cm pieces. Tissue pieces were washed twice in PBS containing 3 mmol/L EDTA for 10 min at 37°C with rotation. To remove EDTA, colon pieces were subsequently washed twice in RPMI-1640 containing 1% FCS, 1 mmol/L EGTA, and 1.5 mmol/L MgCl_2_ for 15 min at 37°C with rotation. Colon pieces were then intensely vortexed, washed with PBS, and digested in RPMI-1640 containing 20% FCS and 100 U/ml collagenase (Sigma-Aldrich) at 37°C for 90 min. Remaining tissue was separated from cells by passing the cell suspension through a 40-mm cell strainer and washing with culture medium.

### Flow Cytometry

Flow cytometry was performed as described previously with several modifications (Xu et al., 2017). All antibodies were purchased from BD Pharmingen. For Th1/Th2/Th17 cells analysis, 2 × 10^6^ of splenocytes or lymphocytes were stimulated at 37°C in 5% CO_2_ for 5 h, in complete RPMI 1640 medium (Gibco, Grand Island, NY) containing 25 ng/ml PMA (Sigma), 0.66 ml/ml Golgistop (BD, San Jose, CA), and 1 mg/ml ionomycin (Sigma). After being surface stained with fluorescently labeled antibodies against surface molecules (CD3-Percp-Cy5.5 and CD4-FITC), cells were fixed and permeabilized with Cytofix/Cytoperm buffer and stained with antibodies against IFN-γ-PE, IL-4-PE, or IL-17-PE, respectively.

For Treg cell analysis, 2 × 10^6^ of splenocytes or lymphocytes were stained with CD4-FITC and CD25-PE-Cy7, after which cells were fixed and permeabilized with fixation-permeabilization buffers (eBioscience) according to the manufacturer's instructions. Subsequently blocked by Fc-receptor (eBioscience, San Diego, CA), cells were stained intracellularly with PE-conjugated anti-Foxp3 antibodies.

Flow cytometry was performed using a BD FACSVerse™ cytometer (BD Biosciences, Heidelberg, Germany) followed by data analysis by FlowJo software (Treestar Inc., San Carlos, CA).

### Splenocyte Preparation and *In vitro* Stimulation

Splenocytes (2 × 10^6^), which were purified from 6-week-old Balb /c mice, were cultured in 12-well round-bottom microtiter culture plates at a volume of 500 μl of RPMI culture medium containing 10% (v/v) heat-inactivated FBS, 1:200 l-glutamine, and 1:100 penicillin-streptomycin and were incubated at 37°C for 48 h. Sodium butyrate (500 μM) was gradient diluted into five different concentration then cocultured with splenic cells for 12 h. The bacteria were harvested and killed by exposure to UV light (Brix et al., 2010). Splenocytes from normal Balb/c mice were stimulated with different doses of microbiome for 12 h, after which cells were collected, and Treg cells were analyzed by FACS.

### Immunofluorescence

Colon tissues from each group were fixed with 4% paraformaldehyde, and then permeabilized with 0.5% Triton X-100 solution for 15 min at indoor temperature. PBS + 2% BSA were used to block unspecific binding sites for 30 min at 4°C. Tissues were stained with rabbit-anti-mouse pho-p65 (Cell Signaling Technology), rabbit anti-mouse pho-p100 (Cell Signaling Technology), or rat anti-mouse Ki-67-APC (BD Biosciences), respectively. Four wash steps with PBS + 0.2% Triton X-100 were performed to remove excess antibody, and then tissues were stained with Alexa Fluor 488-conjugated goat-anti-rabbit IgG. Finally, tissues were washed by PBS four times and analyzed with a microscope (Carl Zeiss). Quantification of fluorescence was analyzed using Image-ProPlus software 6.0 (Media Cybernetics, Silver Spring, Md., USA).

### Statistics Analysis

Statistical analysis was carried out with the SPSS 21.0 software. All data were presented as mean ± SD. The significance of difference between two groups was identified using a *Student's t*-test. Multiple comparisons were made by one-way ANOVA and followed by LSD *post-hoc* test for comparison between each of the two groups. Statistical analyses for microbiota data were described in section Sequence Processing and Bioinformatics Analysis. *P*-values < 0.05 were considered significant. ^*^P < 0.05, ^**^P < 0.01, ^***^P < 0.001. Graphs were generated using the software GraphPad Prism 7.0.

## Conclusions

In summary, we demonstrate that FMT is capable of reinforcing natural defenses and protecting against gastrointestinal disorders during CAC development. Moreover, we provide evidence to show that FMT could enhance anticancer immunity through inducing Treg cells in the microenvironment of tumors. Our data suggest a promising therapeutic strategy for FMT in the treatment of intestinal cancer, although the safety and ethical concern behind FMT treatment still needs further researches (Borody and Khoruts, 2011).

## Data Availability Statement

The datasets generated for this study can be found in Sequence Read Archive (SRA), SUB6299086.

## Ethics Statement

All experiments were performed in strict accordance with the Regulations for the Administration of Affairs Concerning Experimental Animals (1988.11.1). The protocol was approved by the Institutional Animal Care and Use Committee (IACUC) of Nanjing Medical University for the use of laboratory animals.

## Author Contributions

ZW, WH, CL, HC, RL, YN, HS, YLi, XW, and MH carried out the animal experiments. ZW, WH, and ZX participated in analyzing the data. ZX and MJ conceived and designed the experiments. ZW, WH, and ZX drafted the manuscript. YLiu gives valuable suggestions to this manuscript. All authors read and approved the final version of the paper.

### Conflict of Interest

The authors declare that the research was conducted in the absence of any commercial or financial relationships that could be construed as a potential conflict of interest.
