# Supplementary material for: Protective Role of Fecal Microbiota Transplantation on Colitis and Colitis-Associated Colon Cancer in Mice Is Associated With Treg Cells
Source: Front Microbiol. 2019 Nov 12;10:2498. doi: 10.3389/fmicb.2019.02498 (PMC6861520; doi:10.3389/fmicb.2019.02498)
Supplement: Supplementary file 1 [file Table_1.pdf]

Table 1. Sequences of primers used in the study

Gene Symbol	Primer	Primer Sequence (5′ → 3′)
Bacteroidetes	Bac960F	GTTTAATTCGATGATACGCGAG
	Bac1100R	TTAASCCGACACCTCACGG
Firmicutes	Firm934F	GGAGYATGTGGTTTAATTCGAAGCA
	Firm1060R	AGCTGACGACAACCATGCAC
Actinobacteria	Act664F	TGTAGCGGTGGAATGCGC
	Act941R	AATTAAGCCACATGCTCCGCT
Deferribacteres	Defer1115F	CTATTTCCAGTTGCTAACGG
	Defer1265R	GAGHTGCTTCCCTCTGATTATG
Verrucomicrobia	Ver1165F	TCAKGTCAGTATGGCCCTTAT
	Ver1263R	CAGTTTTYAGGATTTCCTCCGCC
Tenericutes	Ten662F	ATGTGTAGCGGTAAAATGCGTAA
	Ten862R	CMTACTTGCGTACGTACTACT
Beta-proteobacteria	Beta979F	AACGCGAAAAACCTTACCTACC
	Beta1130R	TGCCCTTTCGTAGCAACTAGTG
Epsilon-proteobacteria	Epsilon940F	TGCCCTTTCGTAGCAACTAGTG
	Epsilon1129R	CTTACGAAGGCAGTCTCCTTA
Delta-and-Gamma	Gamma877F	GCTAACGCATTAAGTRYCCCG
proteobacteria	Gamma1066R	GCCATGCRGCACCTGTCT
Universal	926F	AAACTCAAAKGAATTGACGG
	1062R	CTCACRRCACGAGCTGAC
Bacterial	27F	AGAGTTTGATCCTGGCTCAG
16S rRNA	1525R	AAGGAGGTGWTCCARCC
TNF-α	Forward	CATCTTCTCAAAATTCGAGTGACAA
	Reverse	TGGGAGTAGACAAGGTACAACCC
IL-10	Forward	ACTTTAAGGGTTACTTGGGTTGC
	Reverse	ATTTTCACAGGGGAGAAATCG
IL-6	Forward	GAGGATACCACTCCCAACAGACC

	Reverse	AAGTGCATCATCGTTGTTCATACA
GAPDH	Forward	GGTGAAGGTCGGTGTGAACG
	Reverse	ACCATGTAGTTGAGGTCAATGAAGG
