# Supplementary material for: Protective Role of Fecal Microbiota Transplantation on Colitis and Colitis-Associated Colon Cancer in Mice Is Associated With Treg Cells
Source: Front Microbiol. 2019 Nov 12;10:2498. doi: 10.3389/fmicb.2019.02498 (PMC6861520; doi:10.3389/fmicb.2019.02498)
Supplement: Supplementary file 2 [file Image_1.pdf]

Supplementary Materials

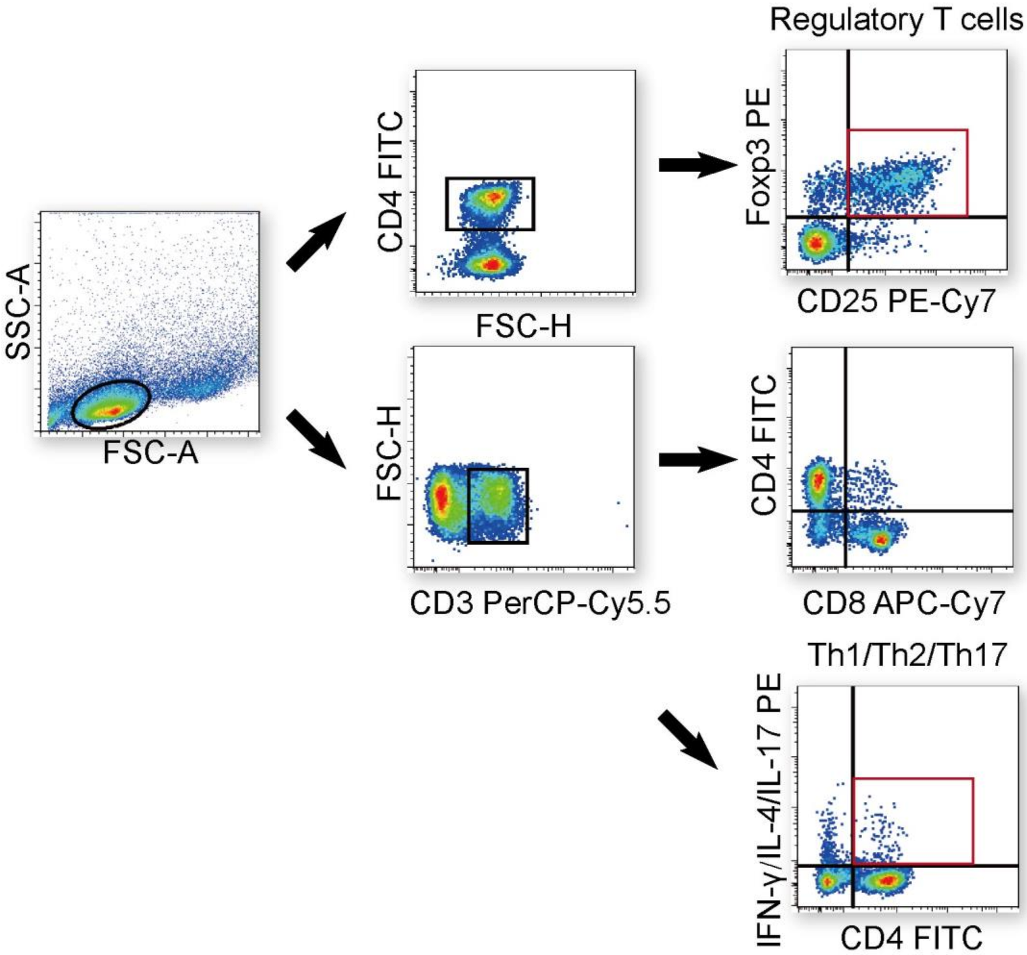

Figure. S1 Gating strategy for FACS.

Gating schemes for analysis of the percentages of $CD4^+CD25^+Foxp3^+$ (Treg) cells, which were gated from $CD4^+$ T cells. And $CD3^+CD4^+$ IFN-γ^+ (Th1), $CD3^+CD4^+$IL-4^+ (Th2), and $CD3^+CD4^+$IL-17^+ (Th17) cells were gated from $CD3^+$ T cells.

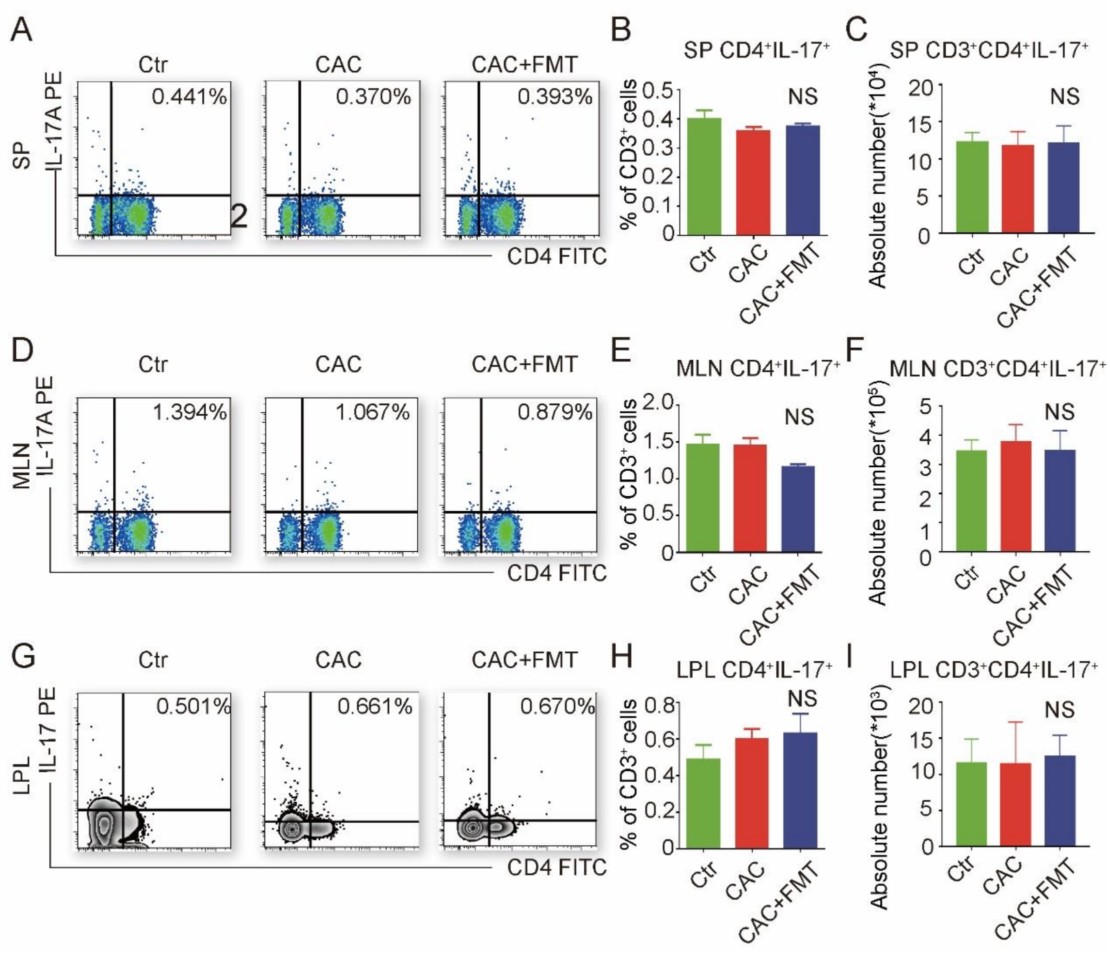

Figure. S2 FACS analysis of helper T helper 17 (Th17) cells in spleen (SP), MLN, and LPL in FMT treated CAC mice.

Single cell suspensions of mouse spleens, MLN, and LPLs from CAC mice treated with or without FMT were prepared. Cells were stained with CD3-Percp-Cy5.5 and CD4-FITC, and then intracellularly stained with PE-conjugated antibodies against IL-17A (PE-IL-17) for FACS analysis of $CD3^+CD4^+IL\text{-}17^+$ (Th17) cells, (**A, D, G)** the representative FACS gates are presented. (**B, E, H**) The proportion of $CD4^+$ in spleen (SP), MLN and LPL of $CD3^+CD4^+IL\text{-}17A^+$ T cells were investigated. (**C, F, I**) The absolute cell numbers of $CD3^+CD4^+IL\text{-}17A^+$ T cells in spleen and MLN were observed. Data are expressed as the mean ± SD of six mice in one representative experiment. All experiments were repeated twice with similar results (ANOVA/LSD).

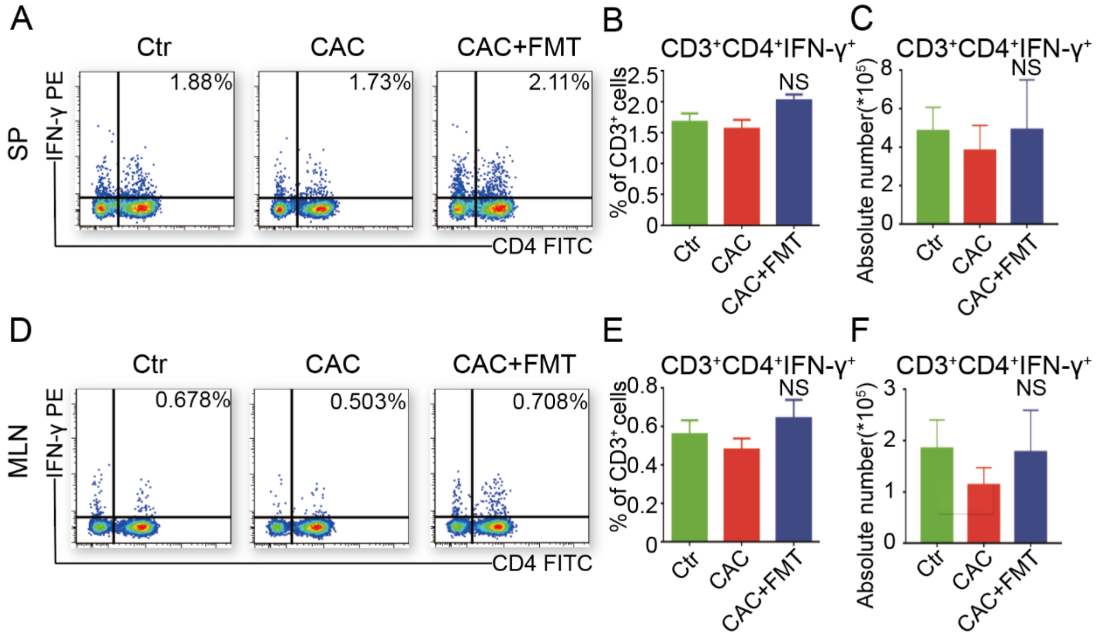

Figure. S3 FACS analysis of helper T helper 1 (Th1) cells in spleen (SP), MLN, of CAC mice treat with or without FMT.

Cells were stained with CD3-Percp-Cy5.5 and CD4-FITC, and then intracellularly stained with PE-conjugated antibodies against IFN-γ (PE-IFN-γ) for FACS analysis of $CD3^{+}CD4^{+}IL\text{-}IFN\text{-}\gamma^{+}$ (Th1) cells, **(A**, **D)** the representative FACS gates are presented. (**B**, **E**) The proportion or (**C**, **F**) absolute cell numbers of Th1 in spleen (SP) and MLN of each group was analyzed. Data are expressed as the mean ± SD of six mice in one representative experiment. All experiments were repeated twice with similar results (ANOVA/LSD).

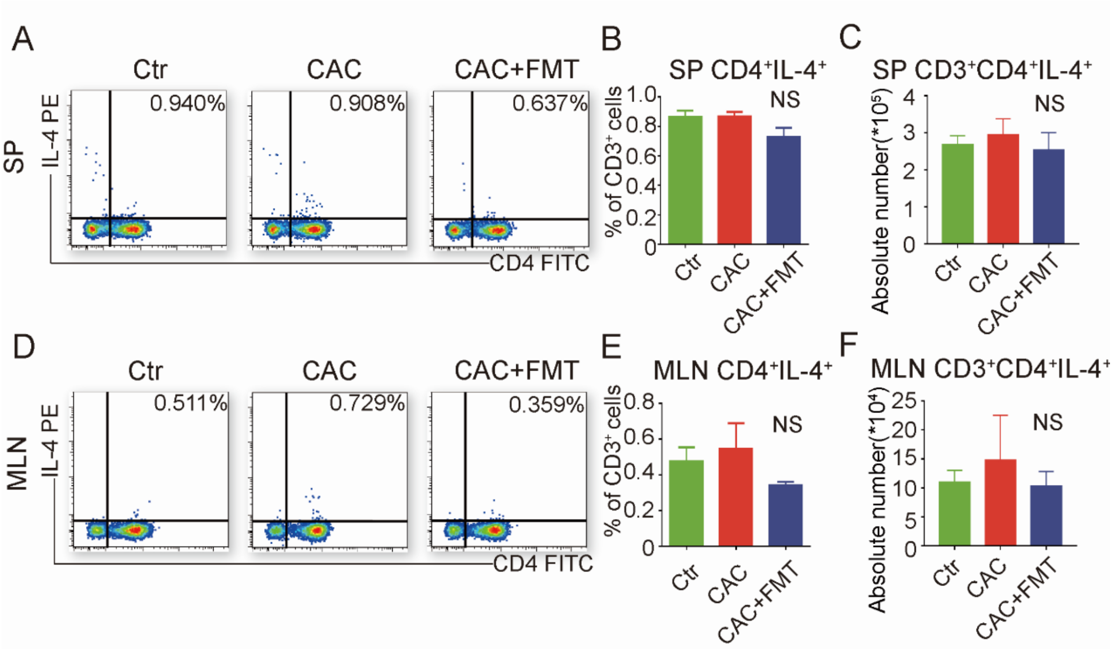

Figure. S4 FACS analysis of helper T helper 2 (Th2) cells in spleen (SP), MLN, of CAC mice treat with or without FMT.

Cells were stained with CD3-Percp-Cy5.5 and CD4-FITC, and then intracellularly stained with PE-conjugated antibodies against IL-4 (PE-IL-4) for FACS analysis of $CD3^{+}CD4^{+}IL\text{-}IL\text{-}4^{+}$ (Th2) cells, (**A**, **D)** the representative FACS gates are presented. (**B, E**) The proportion or (**C, F**) absolute cell numbers of Th2 in spleen (SP) and MLN of each group was analyzed. Data are expressed as the mean ± SD of six mice in one representative experiment. All experiments were repeated twice with similar results (ANOVA/LSD).

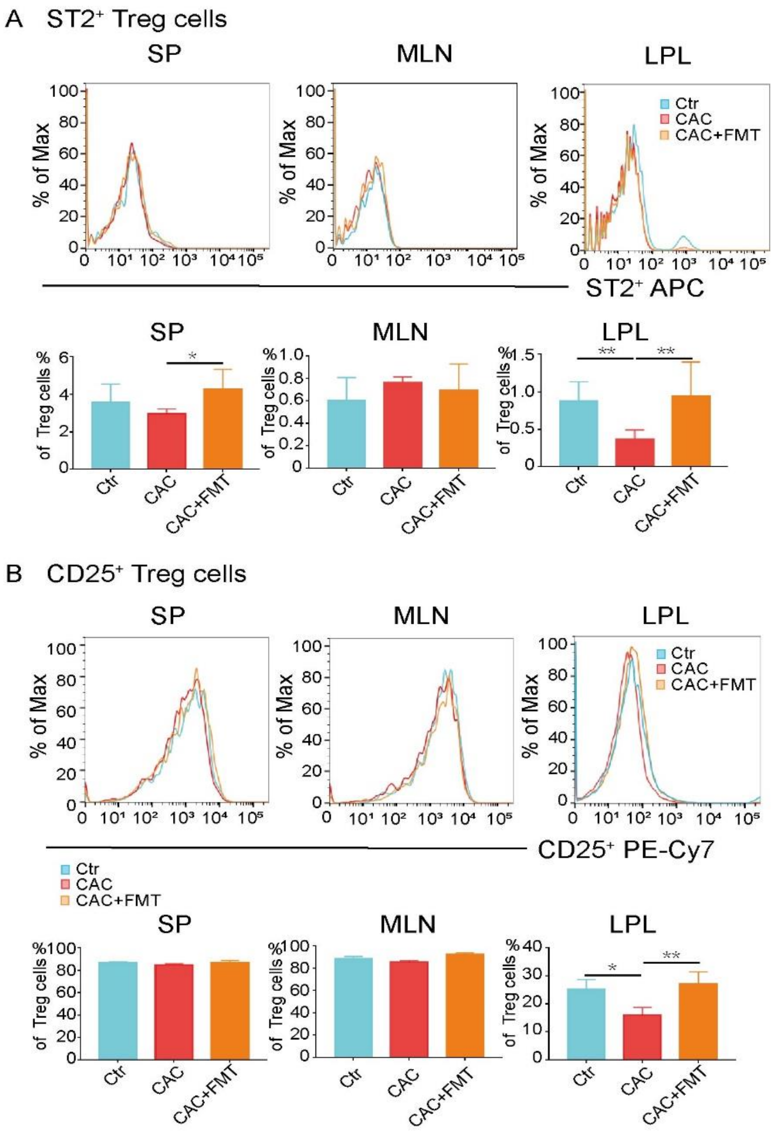

Figure. S5 FACS analysis of ST2$^+$ Treg cell **(A)** and CD25$^+$ Treg cells **(B)** in spleen (SP), MLN, of CAC mice treat with or without FMT. Single-cell suspensions of mouse Lamina propria lymphocyte, mesenteric LN and spleens from each group were prepared. Cells were stained with CD4-FITC, CD25-PE-Cy7 APC-ST2 and then intracellularly stained with PE-conjugated antibodies against Foxp3 and for FACS analysis of regulatory T cells (Treg cells). Representative FACS gates from spleen (a-c, mesenteric LN, and LPL are presented. Data are expressed as the mean ± SD of 6 mice for each group from one representative experiment. All experiments were repeated three times with similar results. *P < 0.05, **P < 0.01, ***P < 0.001 (ANOVA/LSD).

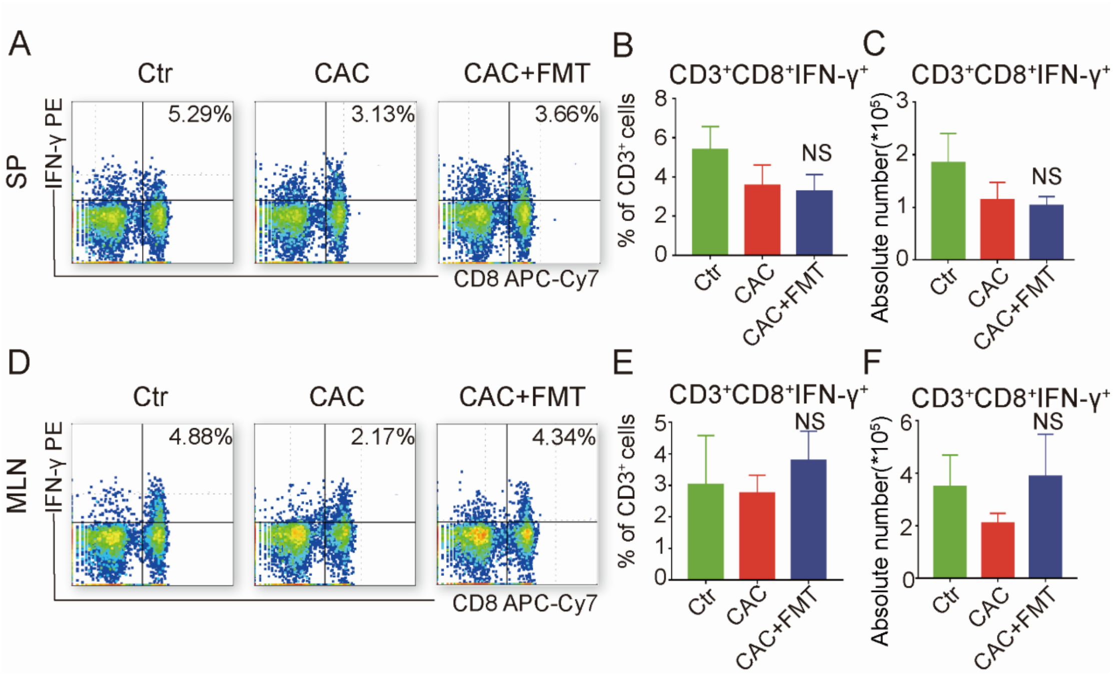

Figure. S6 FACS analysis of cytotoxic T cell (Tc1) cells in spleen (SP), MLN, of CAC mice treat with or without FMT.

Cells were stained with CD3-Percp-Cy5.5 and CD8-FITC, and then intracellularly stained with PE-conjugated antibodies against IFN-γ (PE-IFN-γ) for FACS analysis of $CD3^{+}CD8^{+}IL\text{-}IFN\text{-}\gamma^{+}$ (Tc1) cells, **(a, d)** the representative FACS gates are presented. (**b, e)** The proportion or (**c, f)** absolute cell numbers of Tc1 in spleen (SP) and MLN of each group was analyzed. Data are expressed as the mean ± SD of six mice in one representative experiment. All experiments were repeated twice with similar results, (ANOVA/LSD).

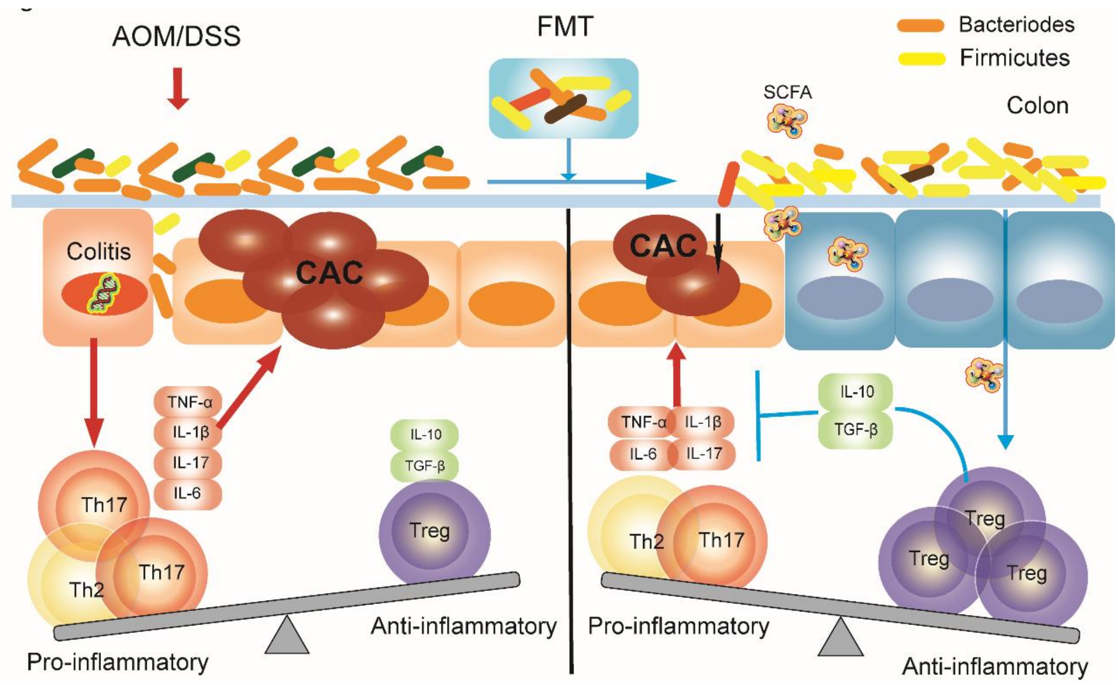

Figure. S7 Diagram of FMT treated CAC and its potential mechanisms.

The current study suggests the possible roles of FMT treatment in regulating immune response and colitis and colitis-associated colon cancer. After FMT treatment, the intestinal microbiota was restored in CAC mice, which promotes the expression of $CD4^{+}CD25^{+}Foxp3^{+}$ regulatory T cells accompanied by attenuating pro-inflammatory response but enhancing anti-inflammatory response in the tumor microenvironment.
